# Association between maternal depression and neonatal outcomes: Evidence from a survey of nationally representative longitudinal studies

**DOI:** 10.3389/fpubh.2022.893518

**Published:** 2022-09-09

**Authors:** Haoran Li, Wei Ning, Ning Zhang, Jingya Zhang, Rongxin He, Ying Mao, Bin Zhu

**Affiliations:** ^1^School of Public Policy and Administration, Xi'an Jiaotong University, Xi'an, China; ^2^Vanke School of Public Health, Tsinghua University, Beijing, China; ^3^School of Public Health and Emergency Management, Southern University of Science and Technology, Shenzhen, China

**Keywords:** birth quality, cohort study, maternal depression, neonatal outcomes, propensity score matching

## Abstract

**Background and aims:**

Maternal depression before and after delivery has dramatically increased in China. Therefore, this study aimed to examine the association between antepartum and postpartum depression and neonatal outcomes.

**Design:**

A population-based retrospective cohort study.

**Setting:**

China.

**Participants:**

Data were obtained from China Family Panel Studies (CFPS). Different mother-child/infant samples were included in this study. Mother in CFPS2012 and CFPS2016 were linked with 1–2-year-old children in CFPS2014 and CFPS2018, respectively. Besides, and mothers in CFPS2012, CFPS2016, and CFPS2018 were linked with 0–1-year-old infants in CFPS2012, CFPS2016, and CFPS2018, respectively.

**Methods:**

Maternal depression was measured using the Center for Epidemiologic Studies Depression Scale. The neonatal outcomes included duration of gestational days, preterm birth, birth weight, birth weight z-score, weight, weight z-score, illness in the past month, and hospitalization in the past year. Propensity score matching was used to balance maternal, family, and infant/child characteristics between the maternal depression and non-maternal depression groups.

**Results:**

Multivariable regression analysis of matched samples estimated that antepartum depression was associated with a shorter duration of gestation by 3.99 days (95% confidence interval [CI] = −7.21, −0.78). The association between antepartum depression and preterm birth, birth weight and birth weight z-score were not statistically significant. Postpartum depression was associated with more episodes of illness in the last month by 0.23 times (95% CI = 0.11, 0.36) and a higher odd of hospitalization in the previous year (OR = 1.59, 95% CI = 1.15, 2.20). The association between postpartum depression and weight or the weight z-score was not significant.

**Conclusion:**

Maternal depression appears to be associated with worse neonatal outcomes.

## Introduction

The quality of the newborn population is an important indicator of a country's social economy and level of medical service. Improving the quality of the newborn population is of great significance for individuals, families, and society (1). Birth quality refers to the quality of the babies before and after they are born. Negative outcomes mainly include sub-healthy births, such as preterm birth, asphyxia, overdue delivery, and low birth weight, which often result in the death of babies or serious diseases and long-term disabilities in children. These factors closely correlate with newborn survival and lifelong health and may bring heavy medical burden to families and society (2, 3). Globally, the preterm birth rate in 2014 was 10.6%, equating to an estimated 14.84 million live preterm births in 2014 (4). The preterm birth rate has increased over the past two decades in almost every country where data are available; 1.1 million babies die from prematurity, and many survivors are disabled or illness like asthma every year (5, 6). Meanwhile, 20.5 million neonates were born with a low birth weight in 2015, and the survivors have a higher risk of stunting, lower intelligence quotient, andd cardiovascular disease later in life than their counterparts (3).

Recently, the proportion of newborns born with sub-health problems in China has increased alarmingly from 25% in 1980 to 65% in 2009. The average number of newborns born with sub-health in China is approximately 1 million every year, which is the main cause of death or disability in newborns in China (7). Monitoring of the quality of the birth population in China started late with small coverage, and the work is also focused on birth defects; thus, few data about sub-healthy births can be collected (2). If the quality of the birth population cannot be fundamentally improved, the number of defective and sub-healthy children will continue to accumulate, and the negative impact will continue to spread among the population, becoming an obstacle to the sustainable development of China in this new situation.

Maternal depression could be considered a potential factor for sub-healthy births. The symptoms of depressed pregnant women are not significantly different from other depressed patients, who may experience loss of appetite, difficulty sleeping, lack of energy, weight loss, and other symptoms (8–10). Moreover, they may smoke and drink more frequently but rarely in China because Chinese culture believes that drug abuse, smoking, and drinking can cause great harm to vulnerable fetuses (11). Chinese scholars surveyed 1,899 pregnant women in 2015 and found that the overall prevalence of prenatal depression was 33.6% (12). Fearful of the effects of antidepressants on their children, women are also less likely to seek medical help or follow medical advice; consequently, they become preoccupied with depressive thoughts, doubt their ability to be a parent, and, more seriously, may self-harm or even commit suicide (8–11). Some studies have demonstrated that depressed pregnant women may have a shorter birth duration (13), a greater risk of preterm birth, delayed fetal development, and a greater risk of low birth weight than non-depressed women (11, 14). Also, some studies showed that depression in pregnant women is not associated with preterm birth, low birth weight, or developmental delay (15, 16).

Postpartum depression has complicated effects on infants or children (17–24), especially in the field of cognitive development; speech and language; visual, motor, emotional/behavioral, and hormonal systems; and infant growth (i.e., body mass index, birth weight, length, and height) (25), and it was also considered as the most significant mental health risk for women during the perinatal period (26, 27). In the published literature in China from 2014 to 2019, the incidence of postpartum depression ranged from 1.66% to 34.8% (28). In terms of infant growth, research has successfully demonstrated the negative effects of postpartum depression in the first 12 months of infant development (25). In both high- and low-income countries, mothers with postpartum depression may have underweight and stunted children (29, 30), especially in infants < 12 months, with a continuous linear growth deficit for up to 5 years (25, 31, 32). However, babies may also gain weight because of early bottle feeding and solid food feeding by depressed mothers (33). Other studies have shown that postpartum depression was not associated with infant growth (34). Additionally, postpartum depression is associated with more episodes of diarrhea and a high risk of infectious and atopic diseases in infants (35).

In summary, according to existing studies, there is no general conclusion regarding the relationship between maternal depression and neonatal outcomes, (15, 16, 34) and this issue deserves further investigation. Because of the lack of attention in China, neither issue has been sufficiently studied. Therefore, in this study, we explored the relationship between antepartum and postpartum depression in mothers of infants in China to compensate for this research gap. Specifically, we aimed to answer two questions. First, does antepartum depression affect neonatal outcomes? Second, does postpartum depression affect neonatal outcomes? Therefore, we proposed the following hypotheses: 1) antepartum depression would have a negative effect on neonatal outcomes and 2) postpartum depression would have a negative effect on neonatal outcomes.

## Methods

### Data and sample

**Figure 1:**
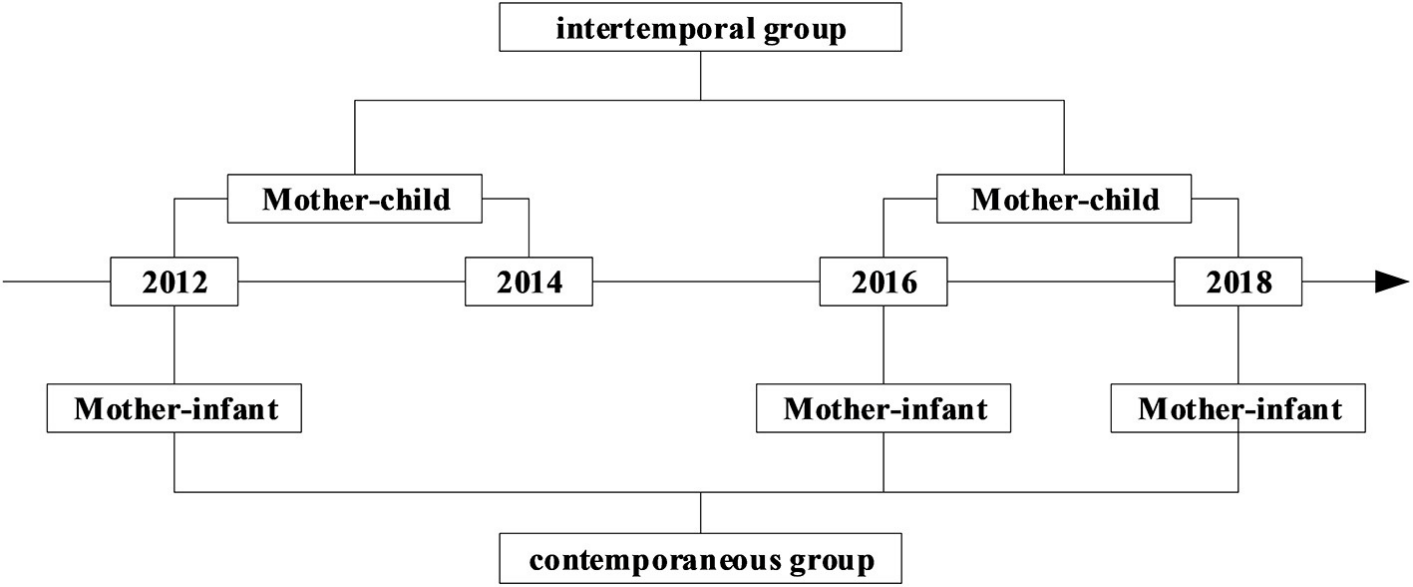
Flow chart of the study groups. The intertemporal group consisted of mothers from 2012 and 2016 who were matched with children from 2014 and 2018, respectively. The contemporaneous group consisted of mother–infant pairs from 2012, 2016, and 2018.

**Figure 2:**
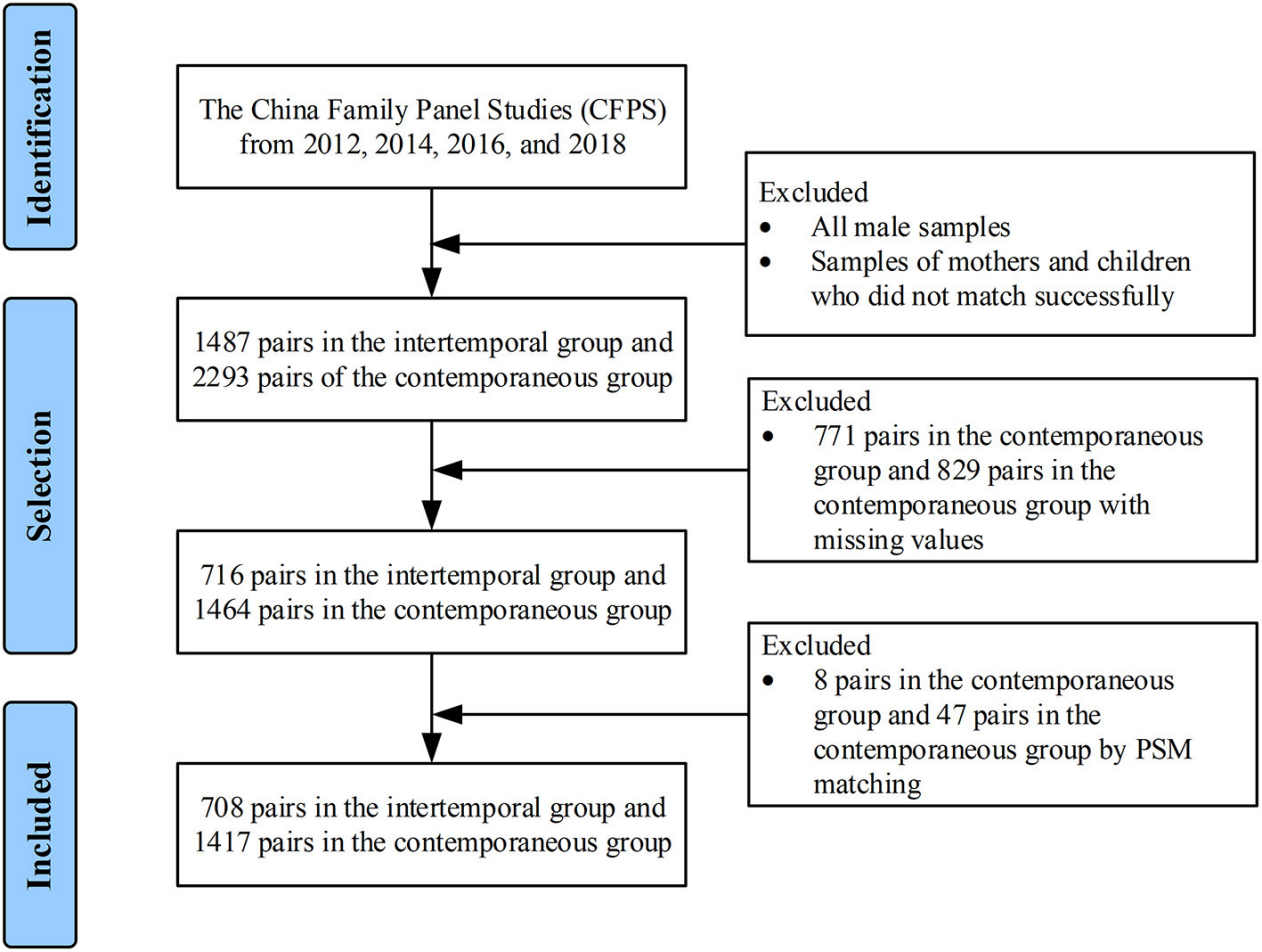
Flow chart of the study participants. Data from the China Family Panel Studies (CFPS) from 2012, 2016, and 2018 were applied.

This study was based on Chinese mother–infant pairs, which were divided into intertemporal and contemporaneous groups. Data from the China Family Panel Studies (CFPS) from 2012, 2016, and 2018 were used in this study. The CFPS, which is a nationally representative sample covering China's 25 provinces/regions and 95% of the population, (36) is implemented by the Chinese Center for Social Science Surveys (ISSS) at Peking University. The CFPS used the Center for Epidemiologic Studies Depression Scale (CESD) to measure depression in adults. Figure 1 illustrates the matching principle. The intertemporal group included mothers in 2012 and 2016 linked with 1–2-year-old children in 2014 and 2018, respectively. So the intertemporal group consisted of 1487 mother-child pairs. The contemporaneous group, including the mothers in 2012, 2016, and 2018, was linked with 0–1-year-old infants in 2012, 2016, and 2018, respectively. So the contemporaneous group consisted of 2293 mother-infant pairs. We further excluded 771 mother-child pairs and 829 mother-infant pairs with missing information on the outcomes and/or covariates required in this study. In total, 716 mother–child pairs and 1464 mother–infant pairs were included in the statistical analysis. Figure 2 presents the detailed selection process.

### Measures

#### Antepartum depression and postpartum depression

The CFPS survey used the Epidemiologic Studies Depression Scale developed by Radloff, which measures the self-rated mental health of respondents using 20 questions (37). Radloff proposed that a score of 16–28 on this scale indicates depressive symptoms, while a score of more than 28 indicates severe depressive symptoms (37). Accordingly, adults who scored more than 28 on the CESD scale of the CFPS2012 and 2016 were defined as “depressed.” In addition, mothers' depression was defined as “antepartum depression” and “postpartum depression” in the intertemporal and contemporaneous groups, respectively.

#### Neonatal outcomes

In the intertemporal group, neonatal outcomes included the duration of gestation (number of days), birth weight (grams), preterm birth (binary indicator for <37 weeks), birth weight z-score (binary indicator for <-2 or >2, calculated using gestational-age-specific birth weight medians and standard deviations by sex). In the contemporaneous group, neonatal outcomes included weight (grams), weight z-score (binary indicator for <-2 or >2, calculated using gestational-age-specific birth weight medians and standard deviations by sex), illness in the past month (number), and hospitalization in the past year (binary indicator).

#### Covariates

The following variables were considered as potential confounding factors in previous studies and were controlled for in this study. The mothers' demographics included age, educational attainment, employment status, and health insurance. Families' characteristics included urban households, region, health expenditure, the Engel coefficient, and poor households. Child demographics included sex.

### Statistical analysis

We used propensity score matching (PSM), a common method used in observational studies, to reduce selection bias. This allowed us to mimic some of the characteristics of a randomized controlled trial by balancing the distribution of observed covariates in the treated and control groups (38–40). First, we used all the covariates described in the Covariates subsection to calculate the propensity score of depression for each pair. Second, children with depressed mothers were matched at a 1:2 ratio to children with normal mothers using nearest-neighbor matching with replacement, the most common implementation of PSM that minimizes bias in subsequent estimations (41). Lastly, we computed standardized differences in covariates between the treated and control groups before and after matching to assess the improvement of balance in the covariates (Supplemental Technical Note 1) (42). If the standardized difference was < 10%, it was considered an indicator of balance (42).

Following PSM, generalized linear mixed regression analyses (Gaussian family model for continuous outcomes and binomial family model for binary outcomes) were used to examine the effect of mothers' antepartum depression and postpartum depression on neonatal outcomes respectively, accounting for the pairing between the treated and control groups in the intertemporal and contemporaneous groups. All the covariates described in the Covariates subsection were adjusted for in the regression analyses. The final sample sizes for the regression analyses following PSM were 708 and 1,417 in the intertemporal and contemporaneous groups, respectively.

In the sensitivity analysis, we used different PSM methods to determine the robustness of the results. In addition to 1:2 nearest-neighbor matching with replacement in the main analysis, we used 1:2 nearest-neighbor matching with replacement and a caliper of 0.001, and 1:4 nearest-neighbor matching with replacement, kernel matching, radius matching, and a caliper of 0.01. In addition, Meanwhile, we divided the samples into subgroups by sex and urban households of infants or children to test for heterogeneity in each group.

All statistical analyses were performed using Stata 15.1 (Stata Corp., LLC). A *p*-value < 0.05 was considered statistically significant.

### Ethics approval

We only used secondary data from the CFPS database which is open for the public. So we did not collect data from the respondents and thus exempted by the Institutional Review Board (IRB) of our university.

## Results

### Propensity score matching

Using PSM, we matched 523 mother–child pairs with antepartum depression and 1,044 mother–child pairs with postpartum depression, which constituted the treated group, with mother–child pairs in the intertemporal group and mother–child pairs in the contemporaneous group, which constituted the matched control group. Supplementary Tables 1, 2 show the descriptive statistics.

**Figure 3:**
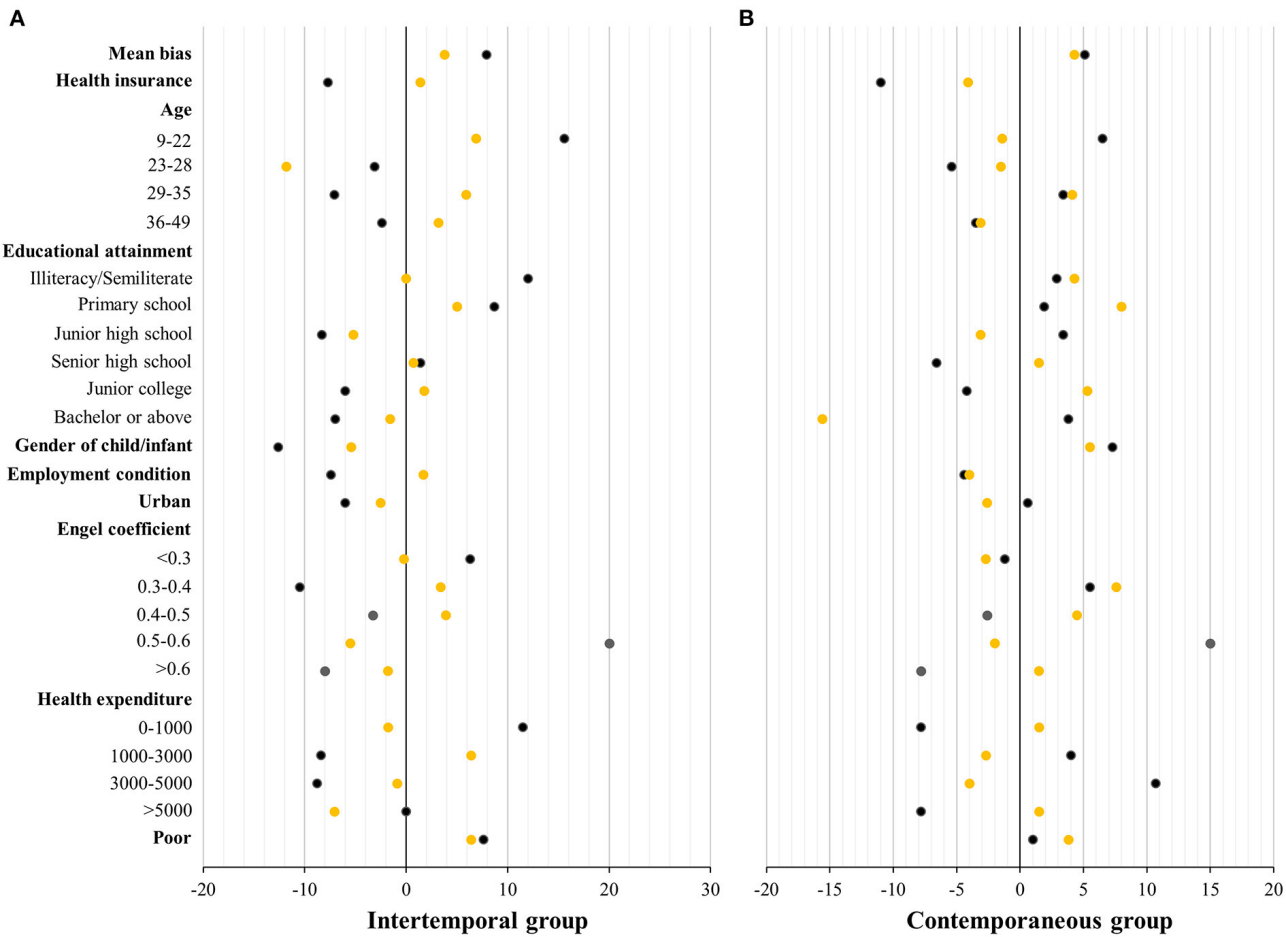
Standardized differences between the treated and control groups before and after propensity score matching. The results of intertemporal group **(A)** and contemporaneous group **(B)** are displayed. The black dots represent standardized differences before matching, and the yellow dots represent standardized differences after matching. The 1:2 nearest-neighbor matching with replacement was used. Details are provided in Supplementary Tables 3, 4.

Figure 3 presents the standardized differences between the treated and control groups before and after 1:2 matching (details are provided in Supplementary Tables 3, 4). In the intertemporal group, some covariates had standardized differences >10% and even up to 20% before matching, but the standardized differences were considerably reduced after matching except for one covariate (age between 23 and 28), which still had a difference >10% and the mean standardized differences reduced from 7.9 to 3.8%, and some covariates had differences reduced to 5% or less. In the contemporaneous group, three covariates had standardized differences >10% before matching, and the standardized differences were reduced to <10% after matching, except for one covariate (junior college), and most standardized differences were reduced to <5%. The mean standardized differences decreased from 5.1 to 4.3%. Therefore, the mean standardized differences were reduced to <5% in both intertemporal and contemporaneous groups. PSM considerably improved comparability between the treated and control groups.

### Association estimation after matching

We first performed descriptive statistics on neonatal outcomes and covariates. Tables 1, 2 show the descriptive statistics of the neonatal outcomes in the intertemporal and contemporaneous groups without regression adjustments. Tables 3, 4 show the descriptive statistics of the descriptive statistics of the covariates for the study sample in the intertemporal and contemporaneous groups.

**Table 1:** Neonatal outcomes between the treated and matched control groups after propensity score matching in the intertemporal group.

**Outcome**	**Treated (*****n*** = **523)**		**Matched control (*****n*** = **185)**		**T test results of the between–group difference**		
	**Mean or %**	**95% CI**	**Mean or %**	**95% CI**	**Mean or %**	**95% CI**	***P*–value**
Duration of gestation, continuous	281.05	279.39, 282.72	285.41	282.80, 288.01	4.35	1.16, 7.54	0.0076
Preterm birth, binary	6.50%	4.38%, 8.62%	3.78%	1.01%, 6.56%	−2.72%	−6.20%, −0.77%	0.1261
Birth weight	3257.46	3211.11, 3303.81	3311.62	3234.53, 3388.72	54.16	−36.10, 144.43	0.2392
Birth weight z–score, continuous	80.31%	76.89%, 83.73%	78.38%	72.39%, 84.37%	−1.93%	−8.68%, −4.82%	0.5753

CI, confidence interval.

**Table 2:** Neonatal outcomes between the treated and matched control groups after propensity score matching in the contemporaneous group.

**Outcome**	**Treated (*****n*** = **1044)**		**Matched control (*****n*** = **373)**		***T*** **-test results of the between–group difference**		
	**Mean or %**	**95% CI**	**Mean or %**	**95% CI**	**Mean or %**	**95% CI**	***P*–value**
Weight, continuous	8975.05	8821.55, 9128.54	8892.55	8615.91, 9168.54	−82.82	−387.86, 222.21	0.5944
Weight z–score, binary	4.02%	2.83%, 5.21%	5.90%	3.50%, 8.30%	1.88%	−0.80%, 4.56%	0.1700
Illness in the last month, continuous	0.72	0.65, 0.78	0.48	0.40, 0.56	−0.24	−0.35, −0.13	0.0000
Hospitalization, binary	23.95%	21.35%, 26.53%	16.09%	12.34%, 19.83%	−7.86%	−12.41%, −3.31%	0.0007

CI, confidence interval.

**Table 3:** Descriptive statistics of the covariates for study samples in the intertemporal group.

**Characteristics**	**Treated** **(*****N*** = **523)**		**Unmatched Control (*****N*** = **193)**		**Matched Control (*****N*** = **185)**	
	***N***	**%**	***N***	**%**	***N***	**%**
***Maternal characteristics***						
**Age**						
16–22	88	16.83	22	11.40	22	11.89
23–28	252	48.18	96	49.74	93	50.27
29–35	143	27.43	59	30.57	54	29.19
36–49	40	7.65	16	8.29	16	8.65
**Medical insurance**	444	84.89	169	87.56	161	87.03
**Education**						
Illiteracy/Semiliterate	59	11.33	15	7.77	15	8.11
Primary school	116	22.18	36	18.65	34	18.38
Junior high school	169	32.31	70	36.27	67	36.22
Senior high school	95	18.16	34	17.62	33	17.84
Junior college	55	10.52	24	12.44	22	11.89
Bachelor or above	29	5.54	14	7.25	14	7.57
**Employment**	268	51.24	106	54.92	101	54.59
***Family characteristics***						
**Urban**	231	44.17	91	47.15	85	45.95
**Poor**	58	11.09	17	8.81	16	8.65
**Health expenditure**						
0–1000	413	78.97	143	74.09	137	74.05
1000–3000	71	13.58	32	16.58	31	16.76
3000–5000	20	3.82	11	5.70	10	5.41
Above 5000	19	3.63	7	3.63	7	3.78
**Engel coefficient**						
Above 0.6	175	33.46	72	37.31	70	37.84
0.5–0.6	66	12.62	13	6.74	13	7.03
0.4–0.5	83	15.87	33	17.10	33	17.84
0.3–0.4	60	11.47	29	15.03	27	14.59
Below 0.3	139	26.58	46	23.83	42	22.70
***Child characteristics***						
**Female**	233	44.55	74	38.34	74	40.00

Treated group included all mother–infant pairs with antepartum depression. Unmatched control group included all mother–infant pairs without antepartum depression. Matched control group included propensity–score–matched mother–infant pairs without antepartum depression.

**Table 4:** Descriptive statistics of the covariates for study samples in the contemporaneous group.

**Characteristics**	**Treated** **(*****N*** = **1044)**		**Unmatched control** **(*****N*** = **420)**		**Matched control** **(*****N*** = **373)**	
	***N***	**%**	***N***	**%**	***N***	**%**
***Maternal characteristics***						
**Age**						
9–22	115	11.02	38	9.05	37	9.92
23–28	501	47.99	213	50.71	186	49.87
29–35	365	34.96	140	33.33	125	33.51
36–49	63	6.03	29	6.90	25	6.70
**Medical insurance**	946	90.61	393	93.57	348	93.30
**Education**						
Illiteracy/Semiliterate	59	5.65	21	5.00	19	5.09
Primary school	156	14.94	60	14.29	51	13.67
Junior high school	380	36.40	146	34.76	128	34.32
Senior high school	201	19.25	92	21.90	85	22.79
Junior college	134	12.84	60	14.29	51	13.67
Bachelor or above	114	10.92	41	9.76	39	10.46
**Employment**	467	44.73	197	46.90	175	46.92
***Family characteristics***						
**Urban**	478	45.79	191	45.48	175	46.92
**Poor**	90	8.62	35	8.33	30	8.04
**Health expenditure**						
0–1000	647	61.97	276	65.71	239	64.08
1000–3000	259	24.81	97	23.10	91	24.40
3000–5000	64	6.13	16	3.81	16	4.29
Above 5000	74	7.09	31	7.38	27	7.27
**Engel coefficient**						
Above 0.6	408	39.08	181	43.10	153	41.02
0.5–0.6	82	7.85	18	4.29	18	4.83
0.4–0.5	147	14.08	63	15.00	56	15.01
0.3–0.4	154	14.75	54	12.86	51	13.67
Below 0.3	253	24.23	104	24.76	95	25.47
***Infant characteristics***						
**Female**	474	45.40	206	49.05	185	49.60

Treated group included all mother–infant pairs with postpartum depression. Unmatched control group included all mother–infant pairs without postpartum depression. Matched control group included propensity–score–matched mother–infant pairs without postpartum depression.

**Figure 4:**
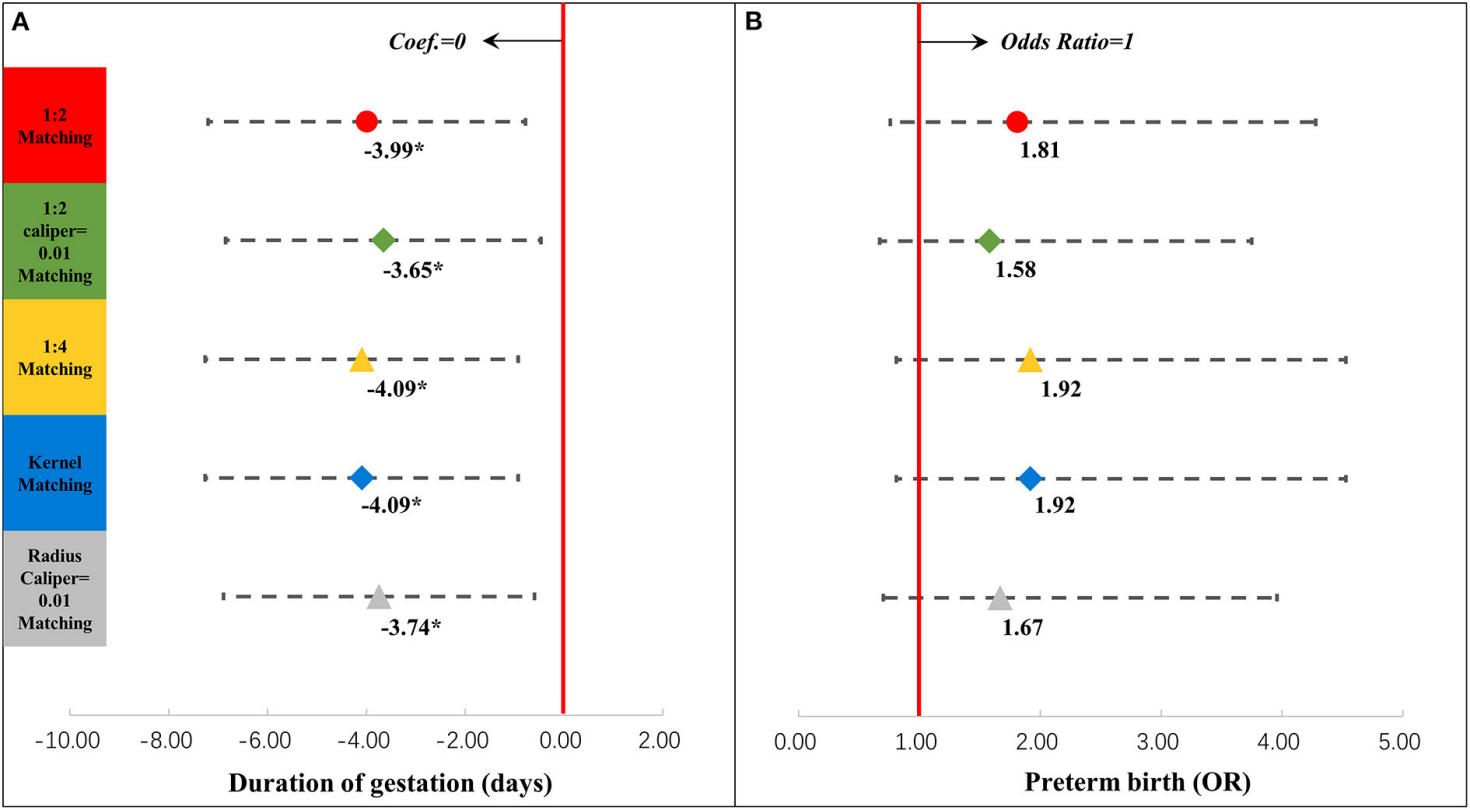
Results of regression analysis of the duration of gestation **(A)** and preterm birth **(B)** after propensity score matching. ^*^P < 0.05, ^**^P < 0.01, ^***^P < 0.001. The results of different propensity score matching analyses are reported too. Dots and lines represent means and 95% confidence intervals for Coef. or ORs estimated from generalized linear mixed regression analyses. Maternal, family, and infant characteristics were also included in the regression analyses but are not reported. Details are provided in Supplementary Table 5. Coef, coefficient; OR, odds ratio.

**Figure 5:**
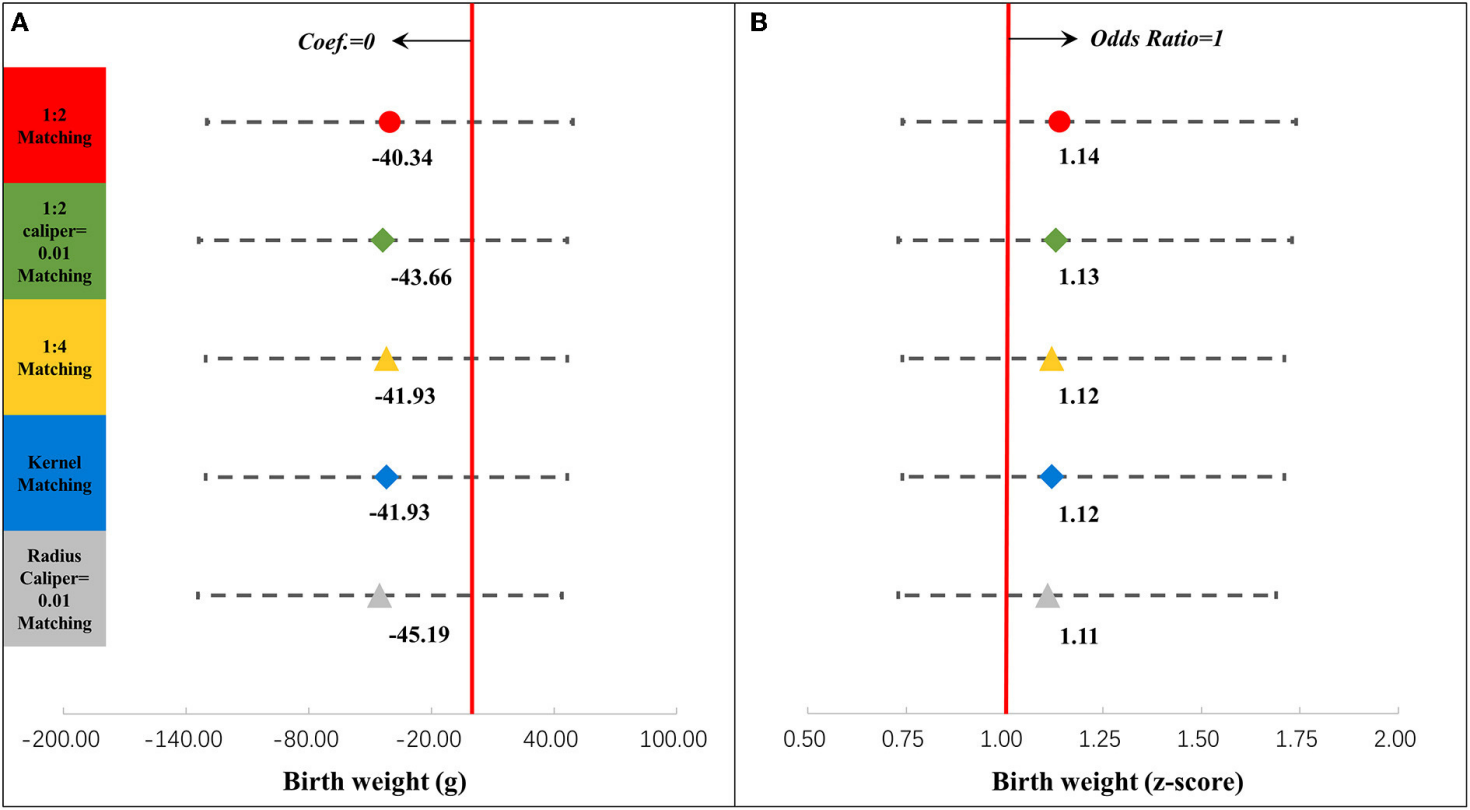
Results of regression analysis of the birth weight **(A)** and birth weight z-score **(B)** after propensity score matching. ^*^P < 0.05, ^**^P < 0.01, ^***^P < 0.001. The results of different propensity score matching analyses are reported too. Dots and lines represent means and 95% confidence intervals for Coef. or ORs estimated from generalized linear mixed regression analyses. Maternal, family, and infant characteristics were also included in the regression analyses but are not reported. Details are shown in Supplementary Table 5. Coef, coefficient; OR, odds ratio.

We then examined the association between antepartum depression and neonatal outcomes in the intertemporal groups. Figure 4 displays the associations between antepartum depression and neonatal outcomes, which were related to gestational age (details are shown in Supplementary Table 5). Antepartum depression was associated with a shorter duration of gestation by 3.99 days (95% confidence interval [CI] = −7.21, −0.78). But the association between antepartum depression and preterm birth was not significant. Figure 5 presents the associations between antepartum depression and neonatal outcomes, which were related to birth weight (details are shown in Supplementary Table 5). The association between antepartum depression and birth weight and birth weight z-score was not significant.

**Figure 6:**
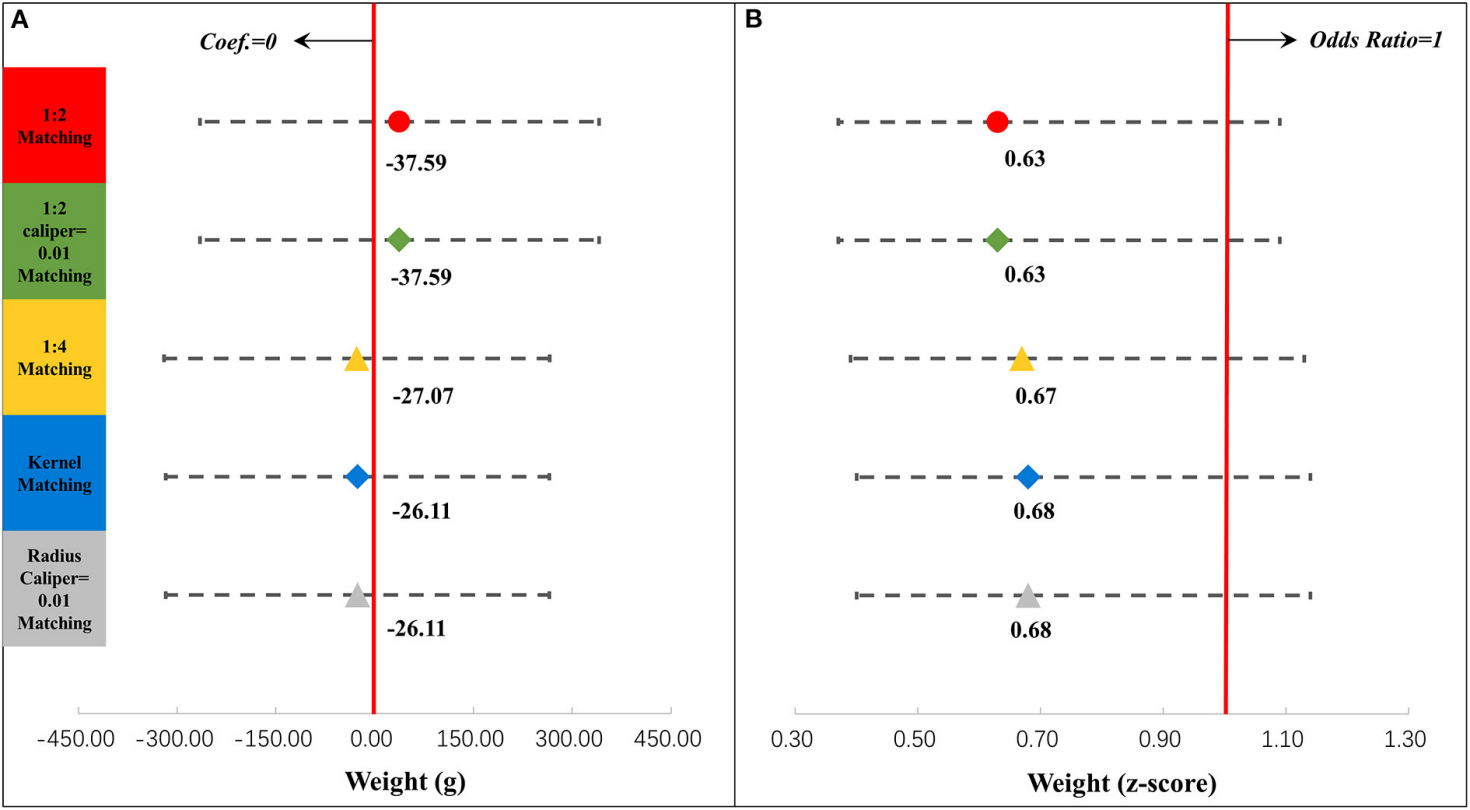
Results of regression analysis of weight **(A)** and the weight z-score **(B)** after propensity score matching. ^*^P < 0.05, ^**^P < 0.01, ^***^P < 0.001. The results of different propensity score matching analyses are reported too. Dots and lines represent means and 95% confidence intervals for Coef. or ORs estimated from generalized linear mixed regression analyses. Maternal, family, and infant characteristics were also included in the regression analyses but are not shown. Details are reported in Supplementary Table 6.

**Figure 7:**
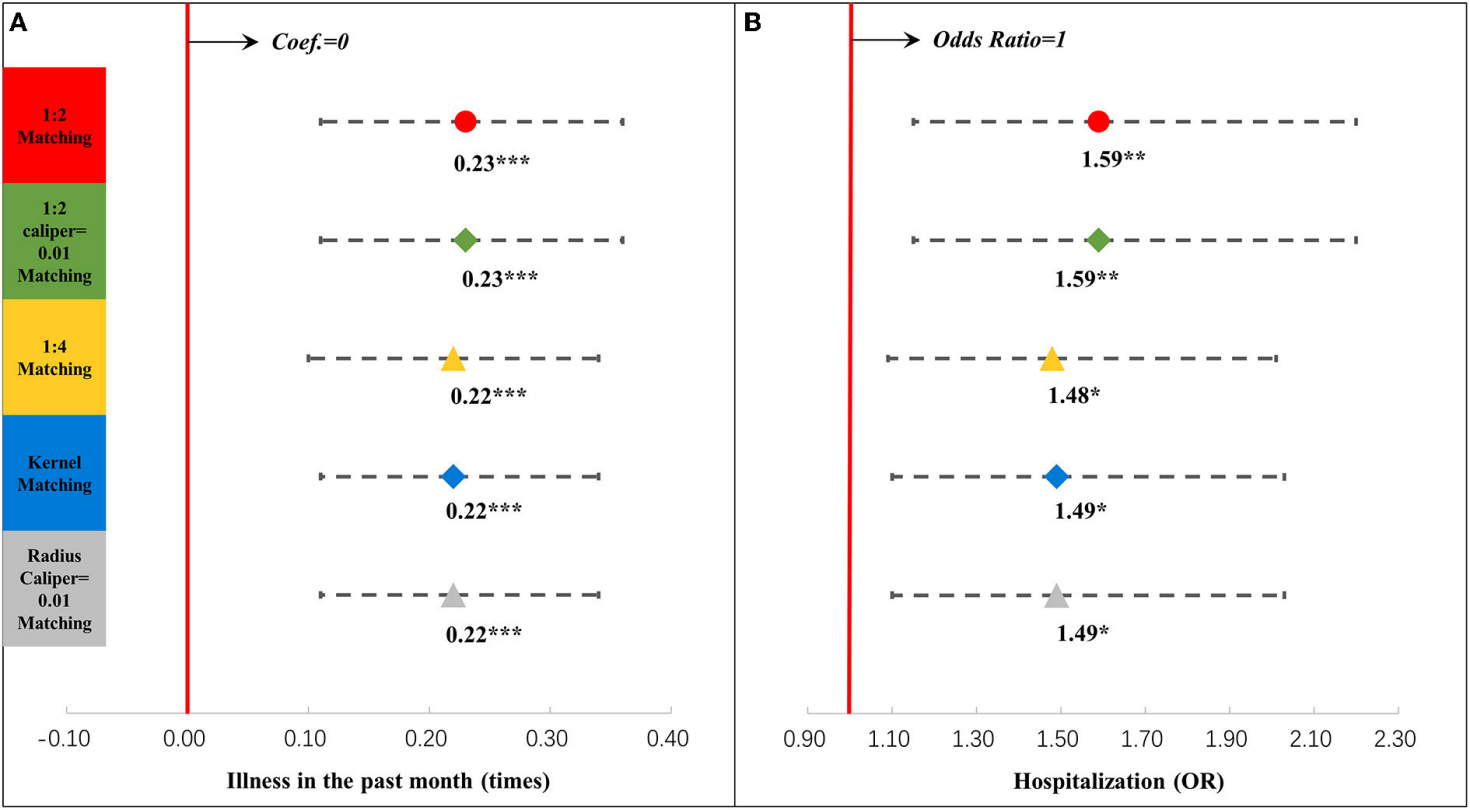
Results of regression analysis of illness in the last month **(A)** and hospitalization **(B)** after propensity score matching. ^*^P < 0.05, ^**^P < 0.01, ^***^P < 0.001. The results of different propensity score matching analyses are shown too. Dots and lines represent means and 95% confidence intervals for Coef. or ORs estimated from generalized linear mixed regression analyses. Maternal, family, and infant characteristics were also included in the regression analyses but are not reported. Details are reported in Supplementary Table 6.

Meanwhile, we examined the association between postpartum depression and neonatal outcomes in the contemporaneous group. Figure 6 shows the associations between postpartum depression and neonatal outcomes, which were related to weight (details are shown in Supplementary Table 6). The association between postpartum depression and weight or the weight z-score was not significant. Figure 7 displays the associations between postpartum depression and neonatal outcomes, which were related to illness (details are shown Supplementary Table 6). Postpartum depression was associated with more episodes of illness in the last month by 0.23 times (95% CI = 0.11, 0.36) and a higher odd of hospitalization in the last year (OR = 1.59, 95% CI = 1.15, 2.20).

We also demonstrated the after-matching results of subgroups by sex and urban households of infants or children in the appendix (details are shown in Supplementary Tables 7–10).

## Discussion

Using a large retrospective cohort in China, this study examined the effects of maternal depression on newborns, which may seriously affect the quality of the birth population. Unlike most previous studies focusing on the effect of antepartum depression or postpartum depression, this study focused on both antepartum depression and postpartum depression. Our PSM analysis revealed that both antepartum and postpartum depression affect newborn health in some ways. Based on the CFPS data, we made several important findings that partially validated our hypotheses.

First, our results showed that antepartum depression was associated with a shorter duration of gestation, but we did not find a significant association between antepartum depression and preterm birth. Larsson et al. found an association between antepartum depressive symptoms and a shorter gestational period of 1 week (13) and other studies have reported no significant association between antepartum depression and preterm birth (14, 16). These studies confirmed our conclusions. But in a previous study from China, Huang et al. reported no link between antepartum depression and preterm birth (43). Furthermore, different from other studies in China (44, 45), the association between antepartum depression and birth weight and birth weight z-scores in our study was not significant. Studies in other countries also suggested no evidence that antepartum depressive symptoms are associated with birth weight (15) or a higher odds of delivering a low-birth-weight infant (16), which was consistent with our conclusion.

It is possible that women with depression may suffer from loss of appetite, difficulty sleeping, lack of energy, weight loss, and other symptoms (8–10), which could biologically affect the fetus. Pregnant women with prenatal depression were also more likely to have pregnancy complications, such as back pain and premature contractions, and were more likely to have mental disorders and fear of childbirth than those without prenatal depression (13). Additionally, mothers with depression are more likely to obtain inadequate medical care or seek medical help than non-depressed mothers (8). But based on our results, all of these potential negative effects of antepartum depression may lead to a shorter duration of gestation, but not so severe as to increase the probability of preterm birth. Meanwhile, our study also proved that antepartum depression was not associated with birth weight.

Second, our study demonstrated that postpartum depression had no significant association with weight or the weight z-score of infants, but postpartum depression was associated with more illnesses in the last month and a higher risk of hospitalization in the previous year. Unlike what we found in our study, evidence from developing countries showed that postpartum depression was associated with early childhood growth, such as being underweight or stunted (30, 35), which was also verified to be true in low-income countries (29). A study on Chinese infants also showed that postpartum depression had a negative effect on the physical development of the baby (46). At the same time, the effect may be persistent, affecting a child's weight gain up to the age of 2 years (32). However, Tomlinson et al. found the same results with us that the association between postpartum and child growth is not significant (34). In addition, many literatures that confirmed that postnatal depressive symptoms can harm a baby's health. A cross-sectional feasibility study in Zambia showed that postpartum depression is associated with more episodes of diarrhea in children. Children with depressed mothers also had more physical problems, such as allergies, asthma, frequent colds, coughs, headaches, and indigestion, than those without non-depressed mothers (47).

Depressive symptoms could also affect maternal care behaviors, such as breastfeeding practices, preparation of appropriate weaning foods, uptake of immunizations, and care-seeking behaviors when children are directly ill (10). Depressed mothers doubt their ability to be a parent (8, 10) and are less likely to provide stimulation and response to their infants than non-depressed mothers (48–50). Consequently, the level of care provided by mothers with depression may put their infants at a higher risk of infection and impaired growth (10). Our findings revealed that maternal depression is a risk factor for the health of newborns and demonstrated the importance of paying attention to women with antepartum depression or postpartum depression in China.

This study has several limitations. First, constrained by the CFPS data, we had to exclude some potential covariates that had too many missing values to keep the sample size as large as possible. We were unable to derive detailed living conditions or childbearing information about mothers, both of which could have potential impacts on neonatal outcomes. Regarding smoking and drinking, as we discussed above, Chinese mothers are less exposed to tobacco and alcohol, but these two factors may have a huge impact on neonatal outcomes (51). Second, we were unable to control for bias from unobserved confounding factors and selection on unobservable confounding factors can still introduce “hidden bias”, although PSM analysis with a range of covariates could balance the measured confounding factors, a limitation common to almost all previous epidemiological studies (52). Third, owing to the nature of the CFPS data, the inability to confirm the exact time of pregnancy and birth could be an issue. However, because of the lack of birth month data, we were able to make a rough intertemporal match between the mother and child to infer when the mother was pregnant, which may have affected our measurements. Lastly, similar to previous findings (25, 31, 32), we found that the effects of postpartum depression on babies can be long-lasting or gradual; some effects do not appear even 5 years later. Because our study only examined 0–1 years old infants after birth, we did not perform a long enough follow-up, so the effects of postpartum depression on a child's growth and development have not been accurately measured. Therefore, it would be worthwhile to investigate and study the long-term effects of maternal prenatal or postnatal depression on children. Future researches are suggested to obtain this.

Despite these limitations, our study has three major strengths. First, we used PSM analysis to improve the validity of comparisons, which have rarely been referred to in previous studies. Second, we used a cohort with CFPS data to verify the influence of maternal depression on newborn health, which compensates for the research gap in China. And the CFPS database allowed us to successfully leverage nationwide, more representative data and reach different conclusions than previous studies. Third, unlike previous studies concentrating only on antepartum depression or postpartum depression, this study verified both.

## Conclusions

This study used CFPS data to determine the effect of antepartum and postpartum depression on neonatal outcomes in China. We found that antepartum depression was associated with a shorter duration of gestation. Postpartum depression was also associated with more frequent illnesses and a higher risk of hospitalization. Our study indicated that maternal depression before and after delivery should be addressed in China. Policies aimed at paying attention to antepartum depression and postpartum depression can not only improve the plight of mothers, but it is also an effective way to improve the quality of birth population. With the various neonatal outcomes, more studies are needed to understand their association with maternal depression in the future.

## Data availability statement

Publicly available datasets were analyzed in this study. This data can be found here: http://www.isss.pku.edu.cn/cfps.

## Author contributions

HL: conceptualization, formal analysis, methodology, formal analysis, and visualization. WN and NZ: formal analysis and visualization. JZ and YM: resources and supervision. RH: supervision. BZ: conceptualization, methodology, visualization, and funding acquisition. All authors contributed to the article and approved the submitted version.

## Funding

This study was funded by the Project of Natural Science Foundation of Guangdong Province (2021A1515110617, PI: Zhu).

## Conflict of interest

The authors declare that the research was conducted in the absence of any commercial or financial relationships that could be construed as a potential conflict of interest.

## Publisher's note

All claims expressed in this article are solely those of the authors and do not necessarily represent those of their affiliated organizations, or those of the publisher, the editors and the reviewers. Any product that may be evaluated in this article, or claim that may be made by its manufacturer, is not guaranteed or endorsed by the publisher.
