# Supplementary material for: Association between maternal depression and neonatal outcomes: Evidence from a survey of nationally representative longitudinal studies
Source: Front Public Health. 2022 Sep 9;10:893518. doi: 10.3389/fpubh.2022.893518 (PMC9500377; doi:10.3389/fpubh.2022.893518)
Supplement: Supplementary file 1 [file Data_Sheet_1.docx]

**Supplemental Materials**

**Technical Note S1** Computation of standardized differences in covariates.

For a dichotomous variable such as tobacco user, the standardized difference is defined as:

$$d=\frac{(\hat{p}_{treated}-\hat{p}_{control} )}{\sqrt{\frac{\hat{p}_{treated}\left( 1-\hat{p}_{treated} \right)+\hat{p}_{control}(1-\hat{p}_{control})}{2}}}$$

Where $\hat{p}_{treated}$ and $\hat{p}_{control}$ denote the percentage of the dichotomous variable taking the value of 1 among infants exposed and unexposed to prenatal cannabis use disorder, respectively.

**Supplementary Table 1** Unadjusted neonatal outcomes between treated and matched control groups after propensity score matching in the intertemporal group.

| **Outcome** | **Treated**  **(*N*=523)** | | **Matched control**  **(*N*=185)** | | **Test on Between-group Difference** |
| --- | --- | --- | --- | --- | --- |
|  | Mean or % | SD | Mean or % | SD | P-value |
| Duration of gestation, continuous | 281.05 | 19.37 | 285.41 | 17.94 | 0.0076 |
| Preterm birth, binary | 6.50% | 24.68% | 3.78% | 19.13% | 0.1261 |
| Birth weight, continuous | 3257.46 | 539.58 | 3311.62 | 531.49 | 0.2392 |
| Birth weight z-score, binary | 80.31% | 39.81% | 78.38% | 41.28% | 0.5753 |

SD: Standard deviation

Notes. Treated group included all mother-infant pairs with antepartum depression. Matched control group included propensity-score-matched mother-infant pairs without antepartum depression.

**Supplementary Table 2** Unadjusted neonatal outcomes between treated and matched control groups after propensity score matching in the contemporaneous group.

| **Outcome** | **Treated**  **(*N*=1044)** | | **Matched control**  **(*N*=373)** | | **Test on Between-group Difference** |
| --- | --- | --- | --- | --- | --- |
|  | Mean or % | SD | Mean or % | SD | P-value |
| Weight, continuous | 8975.05 | 2527.51 | 8892.23 | 2713.91 | 0.5944 |
| Weight z-score, binary | 4.02% | 19.66% | 5.90% | 23.59% | 0.1700 |
| Illness in the past month, continuous | 0.72 | 1.09 | 0.48 | 0.80 | 0.0000 |
| Hospitalization, binary | 23.95% | 42.70% | 16.09% | 36.79% | 0.0007 |

SD: Standard deviation

Notes. Treated group included all mother-infant pairs with postpartum depression. Matched control group included propensity-score-matched mother-infant pairs without postpartum depression.

**Supplementary Table 3** Standardized differences in covariates in the intertemporal group, figure 2 details.

| **Characteristics** | **Treated (N=523) vs. Unmatched Control (N=193)** | | **Treated (N=523) vs. Matched Control (N=185)** | |
| --- | --- | --- | --- | --- |
|  | SD (%) | P-value | SD (%) | P-value |
| ***Maternal Characteristics*** | | | | |
| **Age** |  |  |  |  |
| 9-22 | 15.6 | 0.074 | 6.9 | 0.288 |
| 23-28 | -3.1 | 0.712 | -11.8 | 0.055 |
| 29-35 | -7.1 | 0.395 | 5.9 | 0.324 |
| 36-49 | -2.4 | 0.777 | 3.2 | 0.591 |
| **Medical Insurance** | -7.7 | 0.367 | 1.4 | 0.830 |
| **Education** |  |  |  |  |
| Illiteracy/Semiliterate | 12.0 | 0.172 | 0 | 1.000 |
| Primary school | 8.7 | 0.306 | 5.0 | 0.427 |
| Junior high school | -8.3 | 0.320 | -5.2 | 0.395 |
| Senior high school | 1.4 | 0.866 | 0.7 | 0.904 |
| Junior college | -6.0 | 0.468 | 1.8 | 0.760 |
| Bachelor or above | -7.0 | 0.394 | -1.6 | 0.791 |
| **Employment** | -7.4 | 0.382 | 1.7 | 0.781 |
| ***Family Characteristics*** | | | | |
| **Urban** | -6.0 | 0.477 | -2.5 | 0.686 |
| **Poor** | 7.6 | 0.377 | 6.4 | 0.306 |
| **Health Expenditure** |  |  |  |  |
| 0-1000 | 11.5 | 0.165 | -1.8 | 0.760 |
| 1000-3000 | -8.4 | 0.310 | 6.4 | 0.261 |
| 3000-5000 | -8.8 | 0.275 | -0.9 | 0.874 |
| Above 5000 | 0 | 0.997 | -7.1 | 0.287 |
| **Engel coefficient** |  |  |  |  |
| Above 0.6 | -8.0 | 0.338 | -1.8 | 0.769 |
| 0.5-0.6 | 20.0 | 0.026 | -5.5 | 0.441 |
| 0.4-0.5 | -3.3 | 0.693 | 3.9 | 0.518 |
| 0.3-0.4 | -10.5 | 0.201 | 3.4 | 0.552 |
| Below 0.3 | 6.3 | 0.458 | -0.2 | 0.972 |
| ***Child Characteristics*** | | | | |
| **Female** | -12.6 | 0.137 | -5.4 | 0.383 |
| **Mean bias** | 7.9 | 0.554 | 3.8 | 0.956 |

SD: Standardized differences (in percentage points).

Notes. Treated group included all mother-infant pairs with antepartum depression. Unmatched control group included all mother-infant pairs without antepartum depression. Matched control group included propensity-score-matched mother-infant pairs without antepartum depression. Numbers highlighted in red denote standardized differences ≥ 10%, and numbers highlighted in blue denote standardized differences < 10%.

**Supplementary Table 4** Standardized differences in covariates in the contemporaneous group, figure 2 details.

| **Characteristics** | **Treated (N=1044) vs. Unmatched Control (N=420)** | | **Treated (N=1044) vs. Matched Control (N=373)** | |
| --- | --- | --- | --- | --- |
|  | SD (%) | P-value | SD (%) | P-value |
| ***Maternal Characteristics*** | | | | |
| **Age** |  |  |  |  |
| 9-22 | 6.5 | 0.266 | -1.4 | 0.755 |
| 23-28 | -5.4 | 0.346 | -1.5 | 0.726 |
| 29-35 | 3.4 | 0.554 | 4.1 | 0.344 |
| 36-49 | -3.5 | 0.535 | -3.1 | 0.475 |
| **Medical Insurance** | -11.0 | 0.067 | -4.1 | 0.375 |
| **Education** |  |  |  |  |
| Illiteracy/Semiliterate | 2.9 | 0.620 | 4.3 | 0.323 |
| Primary school | 1.9 | 0.749 | 8.0 | 0.059 |
| Junior high school | 3.4 | 0.555 | -3.1 | 0.483 |
| Senior high school | -6.6 | 0.252 | 1.5 | 0.717 |
| Junior college | -4.2 | 0.459 | 5.3 | 0.200 |
| Bachelor and above | 3.8 | 0.515 | -15.6 | 0.001 |
| **Employment** | -4.4 | 0.450 | -4.0 | 0.357 |
| ***Family Characteristics*** | | | | |
| **Urban** | 0.6 | 0.915 | -2.6 | 0.554 |
| **Poor** | 1.0 | 0.859 | 3.8 | 0.378 |
| **Health Expenditure** |  |  |  |  |
| 0-1000 | -7.8 | 0.180 | 1.5 | 0.736 |
| 1000-3000 | 4.0 | 0.490 | -2.7 | 0.546 |
| 3000-5000 | 10.7 | 0.077 | -4.0 | 0.427 |
| Above 5000 | -1.1 | 0.844 | 5.0 | 0.229 |
| **Engel coefficient** |  |  |  |  |
| Above 0.6 | -8.2 | 0.157 | -5.3 | 0.229 |
| 0.5-0.6 | 15.0 | 0.014 | -2.0 | 0.688 |
| 0.4-0.5 | -2.6 | 0.650 | 4.5 | 0.288 |
| 0.3-0.4 | 5.5 | 0.348 | 7.6 | 0.078 |
| Below 0.3 | -1.2 | 0.832 | -2.7 | 0.543 |
| ***Infant Characteristics*** | | | | |
| **Female** | 7.3 | 0.206 | 5.5 | 0.211 |
| **Mean bias** | 5.1 | 0.402 | 4.3 | 0.117 |

SD: Standardized differences (in percentage points).

Notes. Treated group included all mother-infant pairs with postpartum depression. Unmatched control group included all mother-infant pairs without postpartum depression. Matched control group included propensity-score-matched mother-infant pairs without postpartum depression. Numbers highlighted in red denote standardized differences ≥ 10%, and numbers highlighted in blue denote standardized differences < 10%.

**Supplementary Table 5** Detailed regression results after propensity score matching in the intertemporal group.

| **Outcomes** | **1:2 Matching** | | **1:2 & caliper=0.01**  **Matching** | | **1:4 Matching** | | **Kernel Matching** | | **Radius & Caliper=0.01 Matching** | |
| --- | --- | --- | --- | --- | --- | --- | --- | --- | --- | --- |
|  | Coef./OR (95% CI) | P-value | Coef./OR (95% CI) | P-value | Coef./OR(95% CI) | P-value | Coef./OR (95% CI) | P-value | Coef./OR(95% CI) | P-value |
| Duration of gestation, continuous | -3.99*  (-7.21, -0.78) | 0.015 | -3.65*  (-6.85, -0.46) | 0.025 | -4.09*  (-7.26, -0.93) | 0.011 | -4.09*  (-7.26, -0.93) | 0.011 | -3.74*  (-6.89, -0.60) | 0.020 |
| Preterm birth, binary | 1.81  (0.76,4.28) | 0.178 | 1.58  (0.67,3.75) | 0.300 | 1.92  (0.81,4.53) | 0.139 | 1.92  (0.81,4.53) | 0.139 | 1.67  (0.70,3.96) | 0.246 |
| Birth weight, continuous | -40.34  (-129.92,49.25) | 0.377 | -43.66  (-133.79,46.47) | 0.342 | -41.93  (-130.42,46.56) | 0.352 | -41.93  (-130.42,46.56) | 0.352 | -45.19  (-134.23,43.86) | 0.319 |
| Birth weight z-score, binary | 1.14  (0.74,1.74) | 0.551 | 1.13  (0.73,1.73) | 0.589 | 1.12  (0.74,1.71) | 0.590 | 1.12  (0.74,1.71) | 0.590 | 1.11  (0.73,1.69) | 0.634 |

Notes:

1) *p<.05, **p<.01, ***p<.001.

2) Maternal, family, and infant characteristics were also included in all regressions but not reported.

**Supplementary Table 6** Detailed Regression Results after Propensity Score Matching in the contemporaneous group.

| **Outcomes** | **1:2 Matching** | | **1:2 & caliper=0.01**  **Matching** | | **1:4 Matching** | | **Kernel Matching** | | **Radius & Caliper=0.01 Matching** | |
| --- | --- | --- | --- | --- | --- | --- | --- | --- | --- | --- |
|  | Coef./OR (95% CI) | P-value | Coef./OR (95% CI) | P-value | Coef./OR(95% CI) | P-value | Coef./OR (95% CI) | P-value | Coef./OR(95% CI) | P-value |
| Weight, continuous | 37.59  (-265.45,340.61) | 0.808 | 37.59  (-265.45,340.62) | 0.808 | -27.07  (-319.53,265.40) | 0.856 | -26.11  (-317.59,265.37) | 0.861 | -26.11  (-317.59,265.37) | 0.861 |
| Weight z-score, binary | 0.63  (0.37,1.09) | 0.099 | 0.63  (0.37,1.09) | 0.099 | 0.67  (0.39,1.13) | 0.134 | 0.68  (0.40,1.14) | 0.145 | 0.68  (0.40,1.14) | 0.145 |
| Illness in the past month, continuous | 0.23***  (0.11,0.36) | 0.000 | 0.22***  (0.11,0.36) | 0.001 | 0.22***  (0.10,0.34) | 0.000 | 0.22***  (0.11,0.34) | 0.000 | 0.22***  (0.11,0.34) | 0.000 |
| Hospitalization, binary | 1.59**  (1.15,2.20) | 0.005 | 1.59**  (1.15,2.20) | 0.005 | 1.48*  (1.09,2.01) | 0.012 | 1.49*  (1.10,2.03) | 0.010 | 1.49*  (1.10,2.03) | 0.010 |

Notes:

1) *p<.05, **p<.01, ***p<.001.

2) Maternal, family, and infant characteristics were also included in all regressions but not reported.

**Supplementary Table 7** Detailed Regression Results of male and female group after 1:2 nearest-neighbor matching with replacement in the intertemporal group.

| **Analysis** | **Male** | | **Female** | |
| --- | --- | --- | --- | --- |
|  | Coef./OR (95% CI) | P-value | Coef./OR (95% CI) | P-value |
| Duration of gestation, continuous | -3.73  (-7.87, 0.40) | 0.077 | -4.32  (-9.64, 1.00) | 0.111 |
| Preterm birth, binary | 1.44  (0.48, 4.28) | 0.517 | 3.55  (0.70, 18.02) | 0.127 |
| Birth weight, continuous | -34.44  (-153.40, 84.52) | 0.570 | -74.71  (-217.49, 68.06) | 0.304 |
| Birth weight z-score, binary | 0.944  (0.50, 1.78) | 0.860 | 1.17  (0.61, 2.23) | 0.631 |

Notes:

1) *p<.05, **p<.01, ***p<.001.

2) Maternal, family, and infant characteristics were also included in all regressions but not reported.

**Supplementary Table 8** Detailed Regression Results of male and female group after 1:2 nearest-neighbor matching with replacement in the contemporaneous group.

| **Analysis** | **Male** | | **Female** | |
| --- | --- | --- | --- | --- |
|  | Coef./OR (95% CI) | P-value | Coef./OR (95% CI) | P-value |
| Weight, continuous | -15.48  (-467.28, 436.31) | 0.946 | 26.87  (-364.87, 418.62) | 0.893 |
| Weight z-score, binary | 0.57  (0.27, 1.22) | 0.147 | 1.19  (0.62, 0.96) | 0.312 |
| Illness in the past month, continuous | 0.31  (0.13, 0.49) | 0.001** | 0.83  (0.50, 2.82) | 0.694 |
| Hospitalization, binary | 1.50  (0.98, 0.09) | 0.061 | 1.30  (0.81, 2.08) | 0.282 |

Notes:

1) *p<.05, **p<.01, ***p<.001.

2) Maternal, family, and infant characteristics were also included in all regressions but not reported.

**Supplementary Table 9** Detailed Regression Results of urban and rural group after 1:2 nearest-neighbor matching with replacement in the contemporaneous group.

| **Analysis** | **Urban** | | **Rural** | |
| --- | --- | --- | --- | --- |
|  | Coef./OR (95% CI) | P-value | Coef./OR (95% CI) | P-value |
| Duration of gestation, continuous | -2.53  (-7.43, 2.37) | 0.311 | -5.35  (-9.76, -0.94) | 0.017 |
| Preterm birth, binary | 4.00  (0.82, 19.46) | 0.086 | 1.40  (0.39, 5.03) | 0.604 |
| Birth weight, continuous | -36.45  (-175.7, 102.81) | 0.607 | -38.91  (-159.91, 82.09) | 0.528 |
| Birth weight z-score, binary | 0.82  (0.41, 1.63) | 0.566 | 1.37  (0.77, 2.44) | 0.277 |

Notes:

1) *p<.05, **p<.01, ***p<.001.

2) Maternal, family, and infant characteristics were also included in all regressions but not reported.

**Supplementary Table 10** Detailed Regression Results of urban and rural group after 1:2 nearest-neighbor matching with replacement in the contemporaneous group.

| **Analysis** | **Urban** | | **Rural** | |
| --- | --- | --- | --- | --- |
|  | Coef./OR (95% CI) | P-value | Coef./OR (95% CI) | P-value |
| Weight, continuous | 166.71  (-284.77, 618.20) | 0.469 | -21.94  (-436.56, 392.68) | 0.917 |
| Weight z-score, binary | 3.18  (0.70, 14.43) | 0.134 | 0.57  (0.29, 1.13) | 0.108 |
| Illness in the past month, continuous | 0.27  (0.09, 0.45) | 0.003** | 0.15  (-0.01, 0.32) | 0.072 |
| Hospitalization, binary | 1.67  (0.99, 2.82) | 0.054 | 1.43  (0.94, 2.17) | 0.095 |

Notes:

1) *p<.05, **p<.01, ***p<.001.

2) Maternal, family, and infant characteristics were also included in all regressions but not reported.
